# Influence of Human p53 on Plant Development

**DOI:** 10.1371/journal.pone.0162840

**Published:** 2016-09-20

**Authors:** Huimin Ma, Teng Song, Tianhua Wang, Shui Wang

**Affiliations:** Development Center of Plant Germplasm Resources, College of Life and Environmental Sciences, Shanghai Normal University, Shanghai 200234, China; Indiana University, UNITED STATES

## Abstract

Mammalian p53 is a super tumor suppressor and plays a key role in guarding genome from DNA damage. However, p53 has not been found in plants which do not bear cancer although they constantly expose to ionizing radiation of ultraviolet light. Here we introduced *p53* into the model plant Arabidopsis and examined p53-conferred phenotype in plant. Most strikingly, p53 caused early senescence and fasciation. In plants, fasciation has been shown as a result of the elevated homologous DNA recombination. Consistently, a reporter with overlapping segments of the *GUS* gene (1445) showed that the frequency of homologous recombination was highly induced in *p53*-transgenic plants. In contrast to p53, SUPPRESSOR OF NPR1-1 INDUCIBLE 1 (SNI1), as a negative regulator of homologous recombination in plants, is not present in mammals. Comet assay and clonogenic survival assay demonstrated that SNI1 inhibited DNA damage repair caused by either ionizing radiation or hydroxyurea in human osteosarcoma U2OS cancer cells. RAD51D is a recombinase in homologous recombination and functions downstream of SNI1 in plants. Interestingly, p53 rendered the *sni1* mutants madly branching of inflorescence, a phenotype of fasciation, whereas *rad51d* mutant fully suppressed the p53-induced phenotype, indicating that human p53 action in plant is mediated by the SNI1-RAD51D signaling pathway. The reciprocal species-swap tests of p53 and SNI1 in human and Arabidopsis manifest that these species-specific proteins play a common role in homologous recombination across kingdoms of animals and plants.

## Introduction

Mammalian p53 plays a pivotal role in suppression of tumor. More than half of human cancers bear mutations in the *p53* gene [1]. p53 functions as a node in coordinating the cellular responses to a broad range of stresses with apoptosis, cell cycle arrest, senescence, DNA repair, cell metabolism, or autophagy [2]. p53 not just functions as a transcriptional factor to activate genes during stress responses but also acts independent of transcription especially during apoptosis and DNA damage repair (DDR) [1, 3–6]. p53 has been called “the guardian of the genome” because of its central role in DNA damage repair [2]. In response to DNA damage, the two phosphatidyl inositol 3-kinase-like kinases (PIKKs), ATAXIA TELANGIECTASIA MUTATED (ATM) and ATM-RAD3-RELATED (ATR), activate p53 proteins through phosphorylation on its serine-15 [7–9]. The activated p53 physically interacts with DNA repair proteins including REPLICATION PROTEIN A (RPA), RAD51, RAD54, BREAST CANCER 1 (BRCA1), and BRCA2 [10–18] to non-transcriptionally regulate homologous DNA recombination. p53 is a double-edged sword in regulation of homologous recombination: on the one edge, p53 complexes with RAD51 to inhibit homologous recombination [16, 17, 19, 20]; on the other edge, p53 activates homologous recombination though topoisomerase I [21].

In addition to immunity, SUPPRESSOR OF NPR1-1 INDUCIBLE 1 (SNI1) is a negative regulator of homologous recombination in plant as the frequency of homologous recombination is highly elevated in *sni1* mutants [22, 23]. The *sni1* mutant was first identified as a suppressor of *nonexpressor of pr1 genes 1* (*npr1*) mutant [22]. NPR1 is a master regulator of plant immunity. The *npr1* mutants were notably susceptible to pathogens [24]. To dissect the SNI1 signaling pathway, a genetic approach was employed to isolate *suppressor of sni1* (*ssn*) mutants which restored the wild type phenotype of *sni1* mutant. So far, the characterized SSN proteins are all involved in homologous recombination including RAD51D (SSN1), SSN2 (SWI2/SNF2 and MuDR with SWIM domain), BREAST CANCER 2 (BRCA2, SSN3), RAD51, RAD17 (SSN4), and ATR1 [23, 25–27], suggesting that these positive regulators of homologous recombination function downstream of SNI1 and SNI1 may serve as a brake to attenuate DNA damage response in a proper manner.

The p53 family proteins, including p53, P63 and P73, have been found in Choanozoa and animals. However, they are absent in yeast and plants [28]. In contrast to p53, SNI1 is absent in animals [22, 29]. Previous study showed that overexpression of human *p53* inhibited cell growth of the fission yeast *Schizosaccharomyces pombe* [30] and induced cell death of the budding yeast *Saccharomyces cerevisiae* [31]. In this study, we introduced human *p53* into the model plant Arabidopsis and examined p53-conferred plant phenotype. Since both species-specific proteins p53 and SNI1 function in homologous recombination, we further investigated whether p53 action in plant is mediated by the SNI1 signaling pathway.

## Materials and Methods

### Plant materials

The wild-type background of the mutants used in this study is Columbia (Col-0). Mutants of *sni1* and *rad51d* are as described [22, 23].

### Constructs

To generate *35S*:*p53* for expression of human *p53* in Arabidopsis, the *35S* promoter from pBI121 [32] was amplified by PCR and inserted between BstEII and HindIII of the binary vector pCAMBIA1301 (CAMBIA, Canberra, Australia). Subsequently, the *NOS* terminator was released from pBI121 by SacI and EcoRI digestion and inserted between the corresponding sites of the above *35S* promoter-integrated pCAMBIA1301. Finally, the coding DNA sequence (CDS) of *p53*, obtained from OriGene (Cat. No.: RC200003), was amplified by PCR and inserted between the *35S* promoter and the *NOS* terminator (*35S*:*p53*).

### Measurement of homologous DNA recombination

The frequency of somatic homologous DNA recombination was analyzed using a reporter containing overlapping segments of the *GUS* gene in inverted orientation (line 1445) as described previously [33].

### Microarray analysis

Total RNA of ten-day-old wild type and *p53*-transgenic seedlings was extracted using the RNeasy Plant Mini Kit (Qiagen). The MessageAmp Premier RNA Amplification Kit (Ambion) was used for RNA labeling. Hybridization with the GeneChip Arabidopsis ATH1 Genome Array (Affymetrix), washing, and scanning were performed at the Duke Microarray Facility. Experiments were performed in triplicate. Statistical analysis was performed using GeneSpring GX 11.5 (Agilent).

### Quantitative PCR (qPCR)

Arabidopsis RNA was extracted using TRIzol Reagent (Invitrogen) and measured by NanoDrop 2000 Spectrophotometer (Thermo Fisher). Five μg of RNA was treated with DNase (Ambion TURBO DNA-free Kit, Thermo Fisher). Two μg of DNase-treated RNA was used to synthesize cDNA using the SuperScript III cDNA Synthesis (Invitrogen). The synthesized cDNA, diluted 5 times, was used as templates of qPCR. qPCR was performed using SYBR Green PCR Kit (Roche Applied Science) in Mastercycler ep realplex (Eppendorf). The primers of *RAD51D* used for qPCR are RAD51D-qPCR-F, TTTCGCTATCACGTGACCAT and RAD51D-qPCR-R, TGAAGGCAAGGATGTGTGTT. *UBIQUITIN 5* (*UBQ5*) was used as an internal control which was amplified using primers of UBQ5-qPCR-F, GTAAACGTAGGTGAGTCCA and UBQ5-qPCR-R, GACGCTTCATCTCGTCC.

### Transfection of human U2OS cells

Human osteosarcoma U2OS cancer cells were obtained from the Duke Cell Culture Facility and maintained in McCoy’s 5A medium with 10% fetal bovine serum, 100 units/ml penicillin, and 100 μg/ml streptomycin. The hemagglutinin (HA)-tagged Arabidopsis SNI1 coding DNA sequence (CDS) was integrated into the retroviral vector of pQCXIP (Clontech) for transfection of U2OS cells.

### Immunoblots

Plant tissues were homogenized in liquid nitrogen. Total protein was extracted using a buffer containing 50 mM Tris-HCl (pH 7.5), 150 mM NaCl, 5 mM EDTA, 0.1% Triton X-100, 0.2% Nonidet P-40, and 1% Protease Inhibitor Cocktail (Sigma, P9599). U2OS cancer cells were lysed in RIPA buffer (Sigma, R0278) supplemented with Protease Inhibitor Cocktail (Sigma, P8340). Cell lysates were resolved on sodium dodecyl sulphate–polyacrylamide gel electrophoresis (SDS/PAGE). Western blot analysis was performed as described previously [26]. Anti-α-tubulin antibody (Sigma, T5168) was used as an internal loading control.

### Comet assay

The repair kinetics of ionizing radiation (IR)-induced DNA damage was evaluated by the Alkaline Comet Assay according to the manufacturer's protocol (Trevigen). Briefly, cells were exposed to the indicated doses of IR and harvested at various recovery time points for comet assay. Nuclei were stained with SYBR green and comets were visualized by epifluorescence on a Zeiss microscope.

### Clonogenic survival assay

U2OS cells were plated at 500 cells per well in 6-well plates and pulse-treated with the indicated doses of hydroxyurea (HU, Sigma, H8627) for 24 hours to introduce DNA damage. After treatment, cells were rinsed twice with PBS and allowed to recover in drug-free medium. The cultures were then incubated for 14 days with the medium being changed every 3 days. Colonies were stained with crystal violet (Sigma, C6158), counted, and normalized to untreated control.

### Statistical Analysis

Statistical analysis was performed by one-way ANOVA with Bonferroni post hoc test. The letter above the bar indicates a statistically significant difference between groups at p value < 0.01.

### Accession numbers

The Gene Expression Omnibus (GEO) accession number for the microarray data used in this study is GSE79678 which is available at **http://www.ncbi.nlm.nih.gov/geo/query/acc.cgi?acc=GSE79678**.

## Results

### Human p53 induces early senescence and fasciation in Arabidopsis

**Fig 1:**
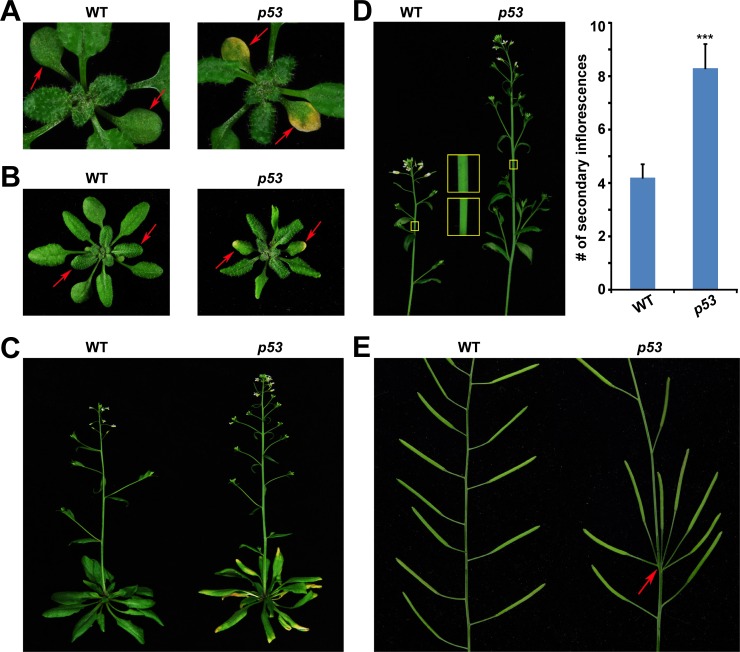
Human p53-conferred phenotype in plant. (A) Leaves of 3-week-old wild type (WT) and *p53*-transgenic plants (*p53*). Arrows indicate cotyledons. (B) Four-week-old WT and *p53* plants. Arrows indicate the first pair of true leaves. (C) Bolting WT and *p53* plants. (D) Left panel: inflorescences of WT and *p53*. Insets show enlarged stems (in yellow box). Right panel: number (#) of secondary inflorescences. Error bars represent standard errors (SEs). ***, p value < 0.001, compared to WT by binomial test. Experiments were carried out in triplicate (n > 30) with similar results. (E) Siliques of WT and *p53*. Arrow indicates clustered (fascinated) siliques.

Ectopic expression of human *p53* under a constitutive promoter, cauliflower mosaic virus (CaMV) *35S* (*35S*:*p53*) [34], in Arabidopsis Columbia (Col-0) resulted in pleiotropic developmental phenotype (Fig 1). We carried out transformation of the *35S*:*p53* construct three times and got 19, 27, and 21 transgenic lines, respectively. p53 proteins were detected by immunoblot in the *p53*-transgenic plants (S1 Fig). All of the *p53*-transgenic lines displayed similar phenotype, except for those died with severe phenotype.

Firstly, p53 induced early senescence which appeared in the cotyledons of three-week-old plants (Fig 1A), in the first pair of true leaves of four-week-old plants (Fig 1B), and most of the leaves of the flowering plants (Fig 1C). In mammals, p53 plays a critical role in senescence which is an irreversible cell-cycle arrest [35]. Overexpression of p53 in human tumor cells triggers senescence [36, 37]. Two cyclin-dependent kinase inhibitors (CKIs) that are often expressed in senescent cells are p21 and p16 [38]. p21 is a crucial transcriptional target of p53 and mediator of p53-dependent senescence [39, 40]. Both p16 and p21 inhibit pRB phosphorylation to activate senescence [38]. Mammalian CKIs are classified into two families: the CDK INTERACTING PROTEIN/KINASE INHIBITORY PROTEIN (Cip/Kip) family, including p21, p27, p57, and the INHIBITORS OF CDK4 (INK4) family, including p16, p15, p18, p19 [41]. Unlike mammals, the involvement of CKIs in plant senescence has not been well understood. CONSTITUTIVE EXPRESSER OF PATHOGENESIS-RELATED GENES 5 (CPR5) is a negative regulator of plant senescence [42]. Recently, SIAMESE (SIM) and SIAMESE-RELATED 1 (SMR1), homologs of mammalian Cip/Kip CKI family proteins, were discovered to function redundantly downstream of CPR5 to activate plant senescence [43]. These data suggest that p53 as well as its mammalian target p21 could activate senescence in both mammals and plants.

Secondly, the *p53*-transgenic plants exhibited fascinated phenotype including thick stems, increased secondary inflorescences, and clustered siliques (Fig 1D and 1E). Fascination is the term used to describe fused or distorted organs along a plant stem [44]. The bolting stems of *p53*-transgenic plants were obviously thicker than those of wild type plants (Fig 1D). The number of secondary inflorescences (lateral branches) of *p53*-transgenic plants was doubled as the average numbers of those of wild type and *p53*-transgenic plants were 4.2 and 8.3, respectively (Fig 1D). Moreover, most of the *p53*-transgenic plants produced clustered (fasciated) siliques (Fig 1E). In Arabidopsis, fasciation has been found in mutants of *fasciata1* (*fas1*), *fasciata2* (*fas2*) and *clavata1* (*clv1*) [44, 45]. CHROMATIN ASSEMBLY FACTOR 1 (CAF-1) is a heterotrimeric complex [46]. Homologs of CAF-1 have been found in yeast, insects, plants, and vertebrates [47]. The Arabidopsis CAF-1 subunits corresponding to the human subunits p150, p60, and p48 are encoded by *FAS1*, *FAS2*, and *MSI1*, respectively [48, 49]. CAF-1 functions in depositing H3/H4 histones onto replicating DNA during S-phase and DNA repair [50–53]. The yeast CHROMATIN ASSEMBLY COMPLEX 1 (CAC1) is the homolog of a subunit of human CAF-1. The frequency of homologous recombination considerably increased in yeast *cac1* mutants [54]. Accordingly, a dominant-negative mutation of a subunit of human CAF-1 resulted in double-strand breaks and activating DNA repair proteins ATM and ATR [55]. ATM was activated by double-strand breaks, whereas ATR was activated by single-strand breaks or stalled replication forks [56]. Like *caf-1* mutants in both yeast and human, the frequency of homologous recombination also dramatically increased (~40-fold) in Arabidopsis *fas1* mutant [47, 57]. Epistatic analysis showed that FAS1 functioned upstream of ATM in homologous recombination as the phenotype of *fas1* mutant was suppressed by *atm* mutant [58]. These data suggest that p53-induced fasciation in plant likely results from the elevated homologous recombination.

### The species-specific p53 and SNI1 play a common role in DNA repair across plants and mammals

**Fig 2:**
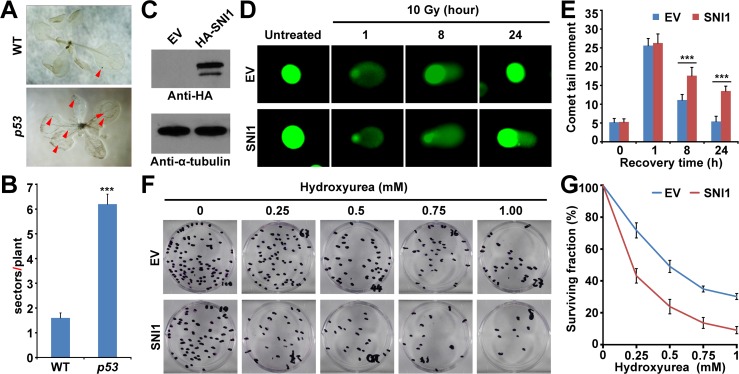
The reciprocal species-swap test of p53 and SNI1 between Arabidopsis and human. (A) Somatic recombination in wild type (WT) and *p53*-transgenic (*p53*) plants is shown in blue sectors by a reporter with overlapping segments of the *GUS* gene (1445). (B) Quantitative result of panel A. Experiments were performed in three *p53*-transgenic lines (n = 50 ~ 100) (S1 Table). The result of line 1 is shown. Error bars represent SEs. ***, p value < 0.001 compared to WT by binomial test. (C) Human osteosarcoma U2OS cancer cells transfected with empty vector (EV) or hemagglutinin (HA)-tagged SNI1 (SNI1). Proteins extracted from the transfected U2OS cancer cells were blotted with anti-HA antibody (abcam, ab1265). Anti-α-tubulin was used as an internal loading control. (D) The comet assay was carried out on the transfected U2OS cancer cells which were treated with 10 Gy of ionizing radiation (IR) and recovered with indicated time. The level of DNA break repair was visualized with the length of comet tail. (E) Images in panel B were analyzed using CometScore software (Tritek) to quantify the comet tail moment of at least 75 cells for each sample. Error bars represent SEs. ***, p value < 0.001, compared to EV by binomial test. Experiments were performed three times with similar results. (F) The transfected U2OS cancer cells were pulse-treated with hydroxyurea (HU) for 24 hours to introduce DNA damage and recovered in drug-free medium. (G) Quantitative results of panel D. After 14 days of culture, colonies were counted and normalized to untreated control. Error bars represent SEs. Experiments were carried out in triplicate.

In mammals, p53 is a double-edged sword which could either inhibit or activate homologous recombination [20, 21]. The p53-induced fasciation indicates that p53 may activate homologous recombination in plant. To quantify the homologous recombination, we introduced the overlapping segments of the *GUS* gene (1445) [33] through crosses into three independent *p53*-transgenic lines. The *GUS* reporter (1445) demonstrated that the frequency of homologous recombination was highly induced by p53 as the sectors per plant of wild type and *p53*-transgenic plants are 1.6 and 6.2, respectively (Fig 2A and 2B and S1 Table). Previously, genetic study revealed that SNI1 was a negative regulator of plant homologous recombination [23]. Intriguingly, comet assay and clonogenic survival assay showed that SNI1 could inhibit DDR caused by either ionizing radiation (IR) or hydroxyurea (HU) in human osteosarcoma U2OS cancer cells (Fig 2C–2G). Immunoblot showed that the SNI1 proteins remained stable with these treatments (S2 Fig). These data demonstrate that both p53 and SNI1 could play a common role in DNA repair across Arabidopsis and human, although they are species-specific proteins.

### Human p53 action in plant is mediated by the SNI1-RAD51D signaling pathway

**Fig 3:**
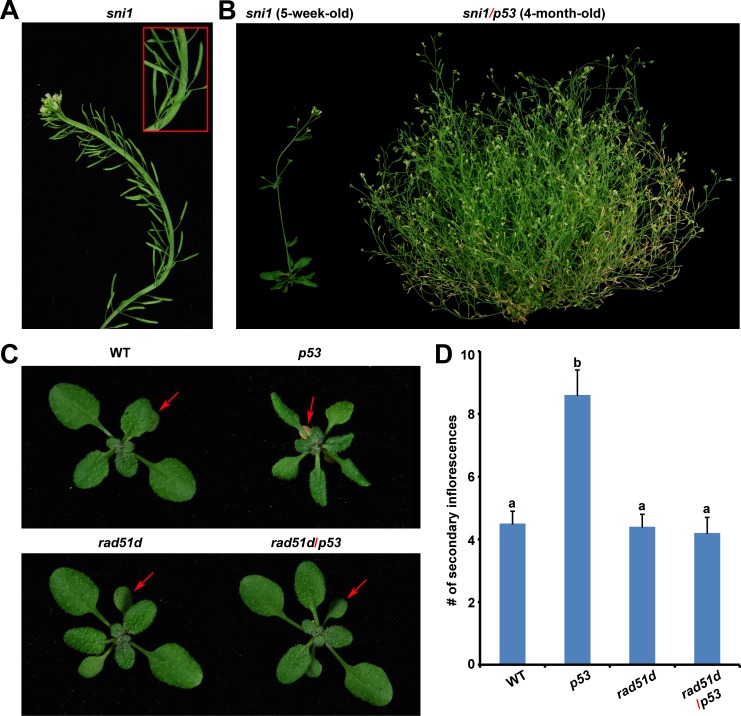
Human p53 acts through the SNI1-RAD51D signaling pathway in plant. (A) Fascinated inflorescence of *sni1* mutant. Inset (box in red): part of the fascinated inflorescence is enlarged. (B) A single plant of five-week-old *sni1* mutant and four-month-old *p53*-transgenic *sni1* mutant (*sni1*/*p53*). (C) Three-week-old WT, *p53*-transgenic (*p53*), *rad51d* and *p53*-transgenic *rad51d* (*rad51d*/*p53*) plants. Arrows indicate cotyledons. (D) Number (#) of secondary inflorescences of WT, *p53*, *rad51d* and *rad51d*/*p53* plants was plotted. The letter above the bar indicates a statistically significant difference between groups at p value < 0.01. Experiments were conducted in triplicate (n > 30) with similar results.

Fasciation was also occasionally observed in *sni1* mutants (Fig 3A), which is consistent with its heightened homologous recombination. Since both *p53*-transgenic plants and *sni1* mutants exhibit fasciation which is presumably associated with homologous recombination, we expect that p53 acts in Arabidopsis through the SNI1 signaling pathway. To test this hypothesis, we introduced *35S*:*p53* construct into *sni1* mutant through crosses with three independent transgenic lines. Surprisingly, *p53*-transgenic *sni1* mutants madly and long lastly (over 4 months) developed secondary inflorescences (Fig 3B), which was a phenotype of fasciation in *fas1*and *fas2* mutants and *p53*-transgenic plants. Since both *fas1* and *sni1* mutants are suppressed by disruptions of DNA repair proteins, we further expect that p53 functions in plant through the SNI1-RAD51D signaling pathway. To testify this possibility, we introduced *35S*:*p53* construct into *rad51d* mutant through crosses with three independent transgenic lines. RAD51D is a paralog of RAD51 which is a key player of homologous recombination. The *rad51d* mutant is the first identified *suppressor of sni1* (*ssn1*) mutant. As expected, *rad51d* fully suppressed p53-induced phenotype including early senescence and fasciation (Fig 3C and 3D), demonstrating that ectopic p53 proteins act in plant through DNA repair protein RAD51D. These data suggest that p53 action is mediated by the SNI1-RAD51D signaling pathway of homologous recombination in plant.

### Regulation of homologous DNA recombination by p53 is independent of transcription in plant

**Fig 4:**
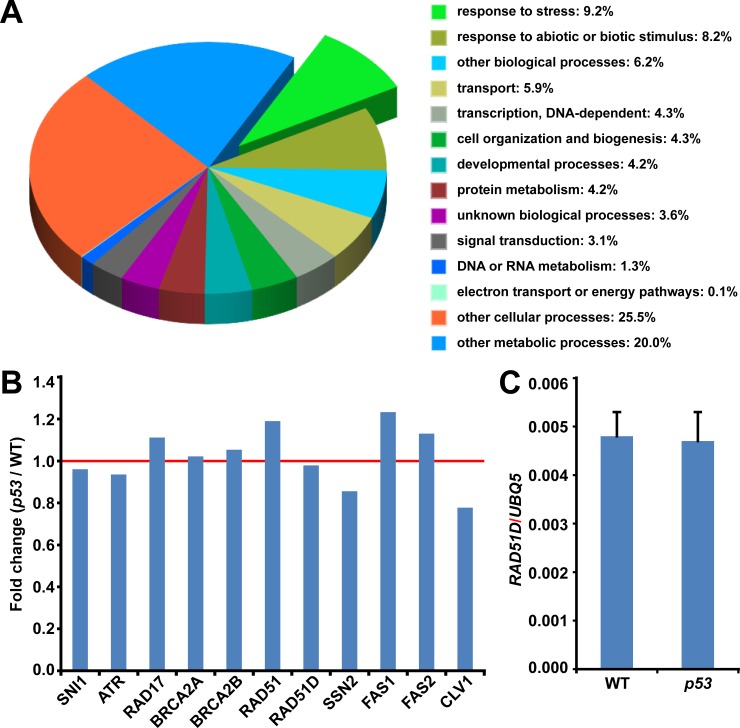
Influence of p53 on plant transcriptome. (A) Gene Ontology (GO) analysis of microarray data. Ten-day-old wild type (WT) and *p53*-transgenic (*p53*) seedlings were used for microarray analysis (GEO accession number: GSE79678). The differential expressed genes (t test, p value < 0.05 and fold change > 2) were analyzed for enriched biological processes by Gene Ontology (GO: https://www.arabidopsis.org/tools/bulk/go/index.jsp). Experiments were performed in triplicate. (B) The expressions of *SNI1*, 7 *SSN*s (*SUPPRESSORS OF SNI1*s) and 3 fascination-associated genes in *p53*-transgenic plants were compared to those in WT plants. The red line indicates the expression with no change. (C) Ten-day-old wild type (WT) and *p53*-transgenic (*p53*) seedlings were used for RNA extraction. *RAD51D* transcripts were quantified by qPCR. *UBQ5* was used as an internal control. Error bars represent SEs. Experiments were conducted in triplicate.

It has been shown that the actions of p53 in mammalian homologous recombination are independent of its transcriptional activity [59, 60]. We adopted a genomic approach to explore how p53 acts in plants. The quality control of microarray is summarized in S2 Table. Gene Ontology (GO) analysis of the differential expressed genes (p value < 0.05 and fold change > 2) induced by ectopic p53 proteins revealed that stress was the most significantly enriched biological process (9.2%) (Fig 4A). However, the expression of *SNI1*, 7 *SSN*s and 3 fascination-associated genes (*FAS1*, *FAS2* and *CLV1*) was not significantly altered (fold change < 1.5) by introduction of p53 in plant (Fig 4B). We adopted quantitative PCR (qPCR) to validate the microarray data. The quality assessments showed that the qPCR assays for *UBQ5* and *RAD51D* were comparable as indicated by the key parameters of the qPCR reaction including slope, precision, amplification and efficiency (S3 Table and S3B–S3D Fig). qPCR analysis validated that p53 did not affect the expression of *RAD51D* (Fig 4C and S3 Table). These data suggest that the influence of human p53 on plant homologous recombination may also be independent of its transactivation as it does in mammals.

## Discussion

Homologous recombination (HR) and nonhomologous end joining (NHEJ) are two main pathways to repair double strand break (DSB) of DNA. The machinery of homologous recombination is highly conserved between animals and plants [61]. As shown in S4A Fig, plants possess most of the key players in homologous recombination [62]. In mammals, the MRE11/RAD50/NBS1 (MRN) complex functions as DSB sensors. RAD17 recruits the MRN complex to the DSB site. In response to DSBs, the MRN complex activates two PIKK kinases: ATM and ATR. ATM is recruited to DSBs by the MRN complex, whereas ATR is recruited by ATR-interacting protein (ATRIP) to RPA-coated single-stranded DNA (ssDNA) [63–66]. ATM and ATR phosphorylate multiple substrates, including p53, and the checkpoint kinases, CHECKPOINT KINASE 1 (CHEK1) and CHECKPOINT KINASE 2 (CHEK2), which inhibits CYCLIN-DEPENDENT KINASE (CDK) activity to allow cell cycle arrest and DNA repair [67]. p53 activates the expression of p21 which is an inhibitor of CDK [1]. Subsequently, de-phosphorylation of BREAST CANCER 2 (BRCA2), which is a substrate of CDK, allows RAD51 to bind DNA and to form nucleofilament that invades a homologous sequence and activates strand exchange to further carry out homologous recombination repair of DSBs [68–70]. Although how plant-specific SNI1 is regulated remains elusive, epistatic analysis suggests that SNI1 functions upstream of the key players of homologous recombination including RAD17, ATR1, BRCA2, RAD51, and RAD51D in plant [23, 26, 27]. Therefore, both p53 and SNI1 play important role in the signaling pathway of homologous recombination.

Our data demonstrate that human p53-conferred plant phenotype including senescence and fasciation is coupled with the elevated homologous recombination which is mediated by the SNI1-RAD51D signaling pathway (Figs 1–3 and S4B Fig). There are at least four possibilities that ectopic p53 proteins could activate homologous recombination. Firstly, p53 is a transcription factor and its excessive accumulation, under the constitutive *35S* promoter, in nucleus could directly cause DNA damage. Secondly, the transcriptional profiling shows that the most significantly enriched biological process is stress which often produces reactive oxygen species (ROS) and indirectly causes DNA damage [71]. Thirdly, p53 may function as a transcription factor to transcriptionally regulate homologous recombination. Fourthly, p53 may complex with protein to non-transcriptionally regulate homologous recombination. It has been discovered that *RADIATION* (*RAD*) genes, including *RAD17* and *RAD51*, are highly upregulated in response to DNA damage in plants [26, 72–75]. As shown in Fig 4B, the expression of *RAD17* and *RAD51* was not significantly affected by the alien p53, ruling out the possibility that human p53-induced homologous recombination in Arabidopsis could be an artifact as a result of the excessive p53-caused DNA damage. Although we cannot exclude with certainty the possibility that p53 transcriptionally regulates homologous recombination through genes other than *SNI1*, 7 *SSN*s and 3 fascination-associated genes, it is likely that p53 acts in plant through interaction with player in the signaling pathway of homologous recombination as it does in mammals [12, 21].

The underlying mechanism of how the SNI1-RAD51D signaling pathway is activated by p53 remains to be investigated. In mammals, p53 is activated through posttranscriptional modifications in response to DNA damage and stress. The activated p53 physically interacts with DNA repair proteins to non-transcriptionally regulate homologous recombination [23, 26, 27]. There is an array of posttranslational modifications including phosphorylation, ubiquitination, acetylation, methylation, sumoylation, neddylation, glycosylation, and ribosylation [76]. It would be fascinating to explore the modifications of p53 protein in response to DNA damage and stress in plants. In this study, our reciprocal species-swap test showed that human p53 caused fasciation through activation of homologous recombination in plants and plant SNI1 inhibited homologous recombination in mammalian cells (Fig 2). It has long been a mystery that plants do not get cancer although they constantly expose to ionizing radiation of ultraviolet light [77, 78]. Our results will stimulate future studies of these species-specific homologous recombination players in a bigger picture which may lead us to better understand why cancers do not bother plants but animals and may help us to better handle human cancers.

## Supporting Information

S1 Fig Detection of p53 proteins in *p53*-transgenic plants. (A) Proteins were extracted from 10-day-old seedlings of wild type (WT) and three lines of *p53*-transgenic plants (*p53*, L1-L3), resolved on sodium dodecyl sulphate–polyacrylamide gel electrophoresis (SDS/PAGE), and immunoblotted with anti-p53 antibody. Anti-α-tubulin was used as an internal loading control. (B) Proteins were extracted from 10-day-old seedlings of wild type (WT) and *p53*-transgenic (L1: line1) WT plants, *sni1*mutant, *rad51d* mutant, and *GUS* (1445) reporter. Immunoblot was performed as panel A.
(TIF)

S2 Fig Detection of SNI1 proteins in *SNI1*-transfected human osteosarcoma U2OS cancer cells. The transfected U2OS cancer cells were treated with 10 Gy of ionizing radiation (IR) or hydroxyurea (HU). Proteins extracted from the transfected transfected U2OS cancer cells were blotted with anti-HA antibody (abcam, ab1265). Anti-α-tubulin was used as an internal loading control.
(TIF)

S3 Fig Quality assay of RNA and qPCR. (A) Total RNA was extracted from ten-day-old wild type and *p53*-transgenic seedlings. The A260/A280 ratio of the total RNA was about 2.0 as measured on NanoDrop 2000 Spectrophotometer. The quality of RNA was further assessed by agarose gel electrophoresis. The 28S/18S ratio was about 2.0, indicating that the isolated RNAs were not degraded. (B) The qPCR products of *UBQ5* and *RAD51D* were viewed by agarose gel electrophoresis. The qPCR product size of *UBQ5* and *RAD51D* is about 250 bp and 100 bp, respectively. M, DNA marker. (C) The melting curves from qPCR analysis of *UBQ5* and *RAD51D*. There was only one peak appeared in the melting curves of qPCR analysis in both *UBQ5* and *RAD51D*, which was consistent with the agarose gel electrophoresis of qPCR product in panel B. (D) Serial dilutions of reverse transcription product (cDNA) were used for the qPCR quality assay of *UBQ5* and *RAD51D*. C_t_, cycle threshold.
(TIF)

S4 Fig The role of p53 and SNI1 in homologous recombination. (A) The signaling pathway of DNA damage repair through homologous recombination in mammals and plants. In mammals, the MRE11/RAD50/NBS1 (MRN) complex functions as double strand brake (DSB) sensors. RAD17 recruits the MRN complex to the DSB site. In response to DNA damage, two phosphoinositide 3-kinase-like kinases, ATM and ATR, are activated by the MRN complex to phosphorylate the transcription factor p53. p21, a target of p53, is an inhibitor of cyclin-dependent kinase (CDK). KIP-RELATED PROTEIN (KRP) is the homolog of p21 in plant. CDK phosphorylates BREAST CANCER 2 (BRCA2) which is a mediator of the recombinase RAD51. BRCA2 first loads RAD51 on the DSB site. After the DSB is repaired by RAD51, BRCA2 is then phosphorylated by CDK to remove RAD51 from DNA. Plants possess the homologs of these DNA repair proteins, except for p53. On the contrary, mammals do not bear SNI1. SNI1 is a negative regulator of homologous recombination in plants. Although genetic study reveals that SNI1 functions upstream of ATR, RAD17, BRCA2, and RAD51, how SNI1 is regulated remains unknown. (B) A proposed model of p53 action in plant. p53 induces homologous recombination through the SNI1-RAD51D signaling pathway in plant, leading to senescence and fasciation.
(TIF)

S1 Table The sectors of recombined *GUS* reporter (1445) in wild type and three lines of *p53*-transgenic plants. (DOCX)

S2 Table The quality control summary of microarray. (DOCX)

S3 Table Quality assay for reverse transcription and qPCR. (DOCX)
