# Supplementary figures and images for: Influence of Human p53 on Plant Development
Source: PLoS One. 2016 Sep 20;11(9):e0162840. doi: 10.1371/journal.pone.0162840 (PMC5029891; doi:10.1371/journal.pone.0162840)

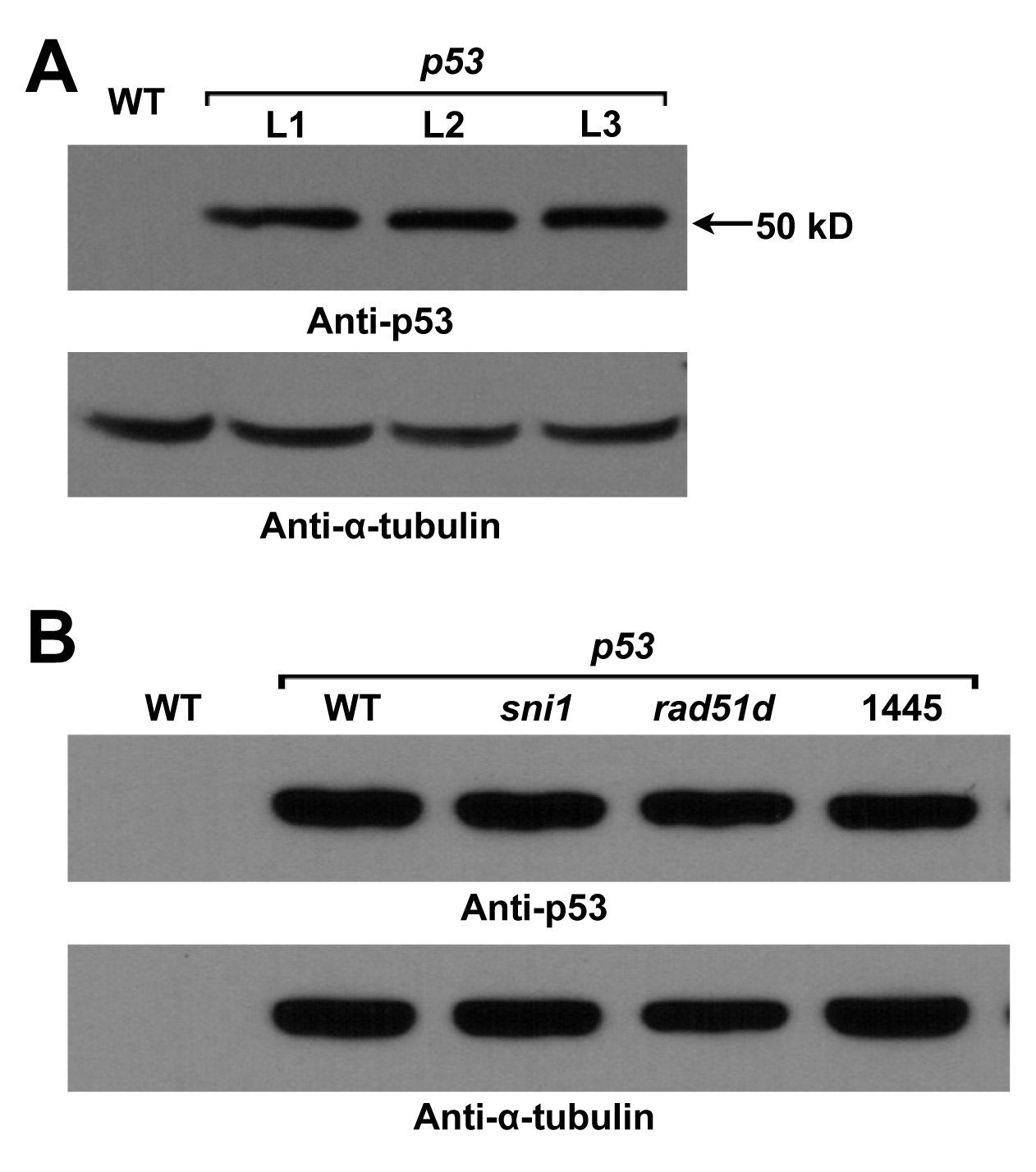

Supplement: S1 Fig — (A) Proteins were extracted from 10-day-old seedlings of wild type (WT) and three lines of p53-transgenic plants (p53, L1-L3), resolved on sodium dodecyl sulphate–polyacrylamide gel electrophoresis (SDS/PAGE), and immunoblotted with anti-p53 antibody. Anti-α-tubulin was used as an internal loading control. (B) Proteins were extracted from 10-day-old seedlings of wild type (WT) and p53-transgenic (L1: line1) WT plants, sni1mutant, rad51d mutant, and GUS (1445) reporter. Immunoblot was performed as panel A. (TIF) [file pone.0162840.s001.tif]

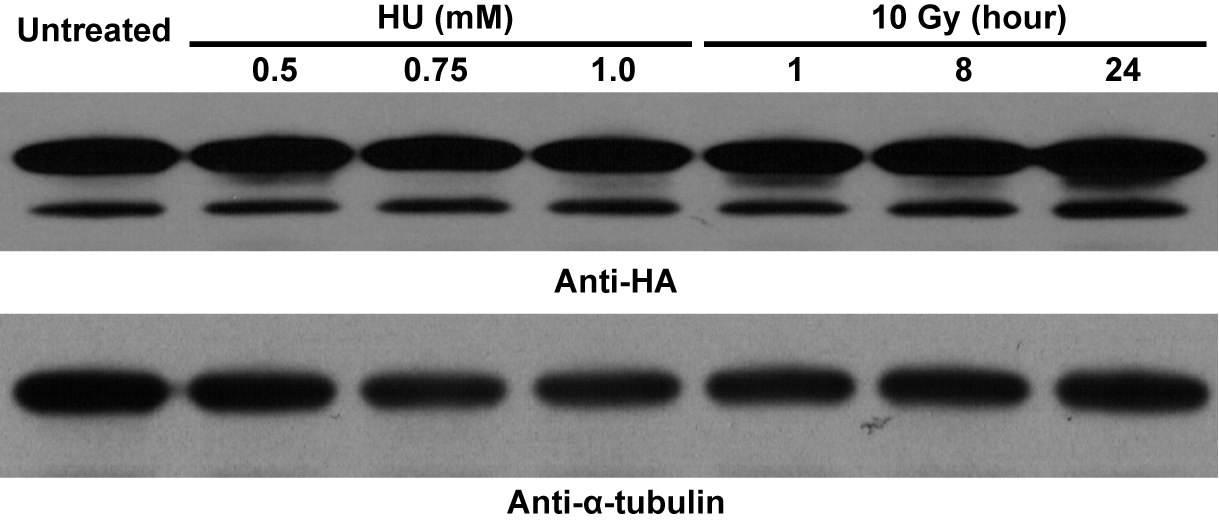

Supplement: S2 Fig — The transfected U2OS cancer cells were treated with 10 Gy of ionizing radiation (IR) or hydroxyurea (HU). Proteins extracted from the transfected transfected U2OS cancer cells were blotted with anti-HA antibody (abcam, ab1265). Anti-α-tubulin was used as an internal loading control. (TIF) [file pone.0162840.s002.tif]

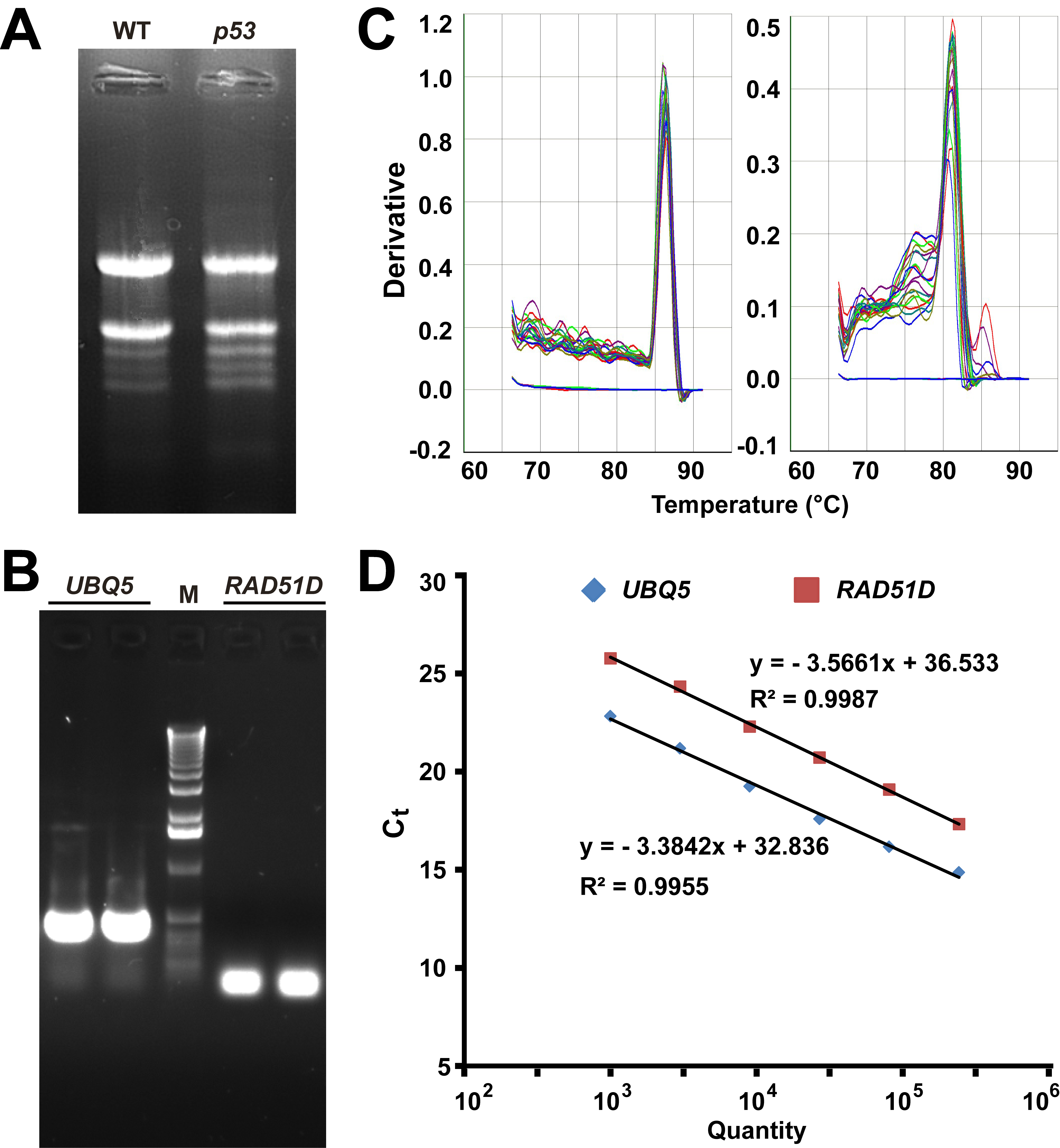

Supplement: S3 Fig — (A) Total RNA was extracted from ten-day-old wild type and p53-transgenic seedlings. The A260/A280 ratio of the total RNA was about 2.0 as measured on NanoDrop 2000 Spectrophotometer. The quality of RNA was further assessed by agarose gel electrophoresis. The 28S/18S ratio was about 2.0, indicating that the isolated RNAs were not degraded. (B) The qPCR products of UBQ5 and RAD51D were viewed by agarose gel electrophoresis. The qPCR product size of UBQ5 and RAD51D is about 250 bp and 100 bp, respectively. M, DNA marker. (C) The melting curves from qPCR analysis of UBQ5 and RAD51D. There was only one peak appeared in the melting curves of qPCR analysis in both UBQ5 and RAD51D, which was consistent with the agarose gel electrophoresis of qPCR product in panel B. (D) Serial dilutions of reverse transcription product (cDNA) were used for the qPCR quality assay of UBQ5 and RAD51D. Ct, cycle threshold. (TIF) [file pone.0162840.s003.tif]

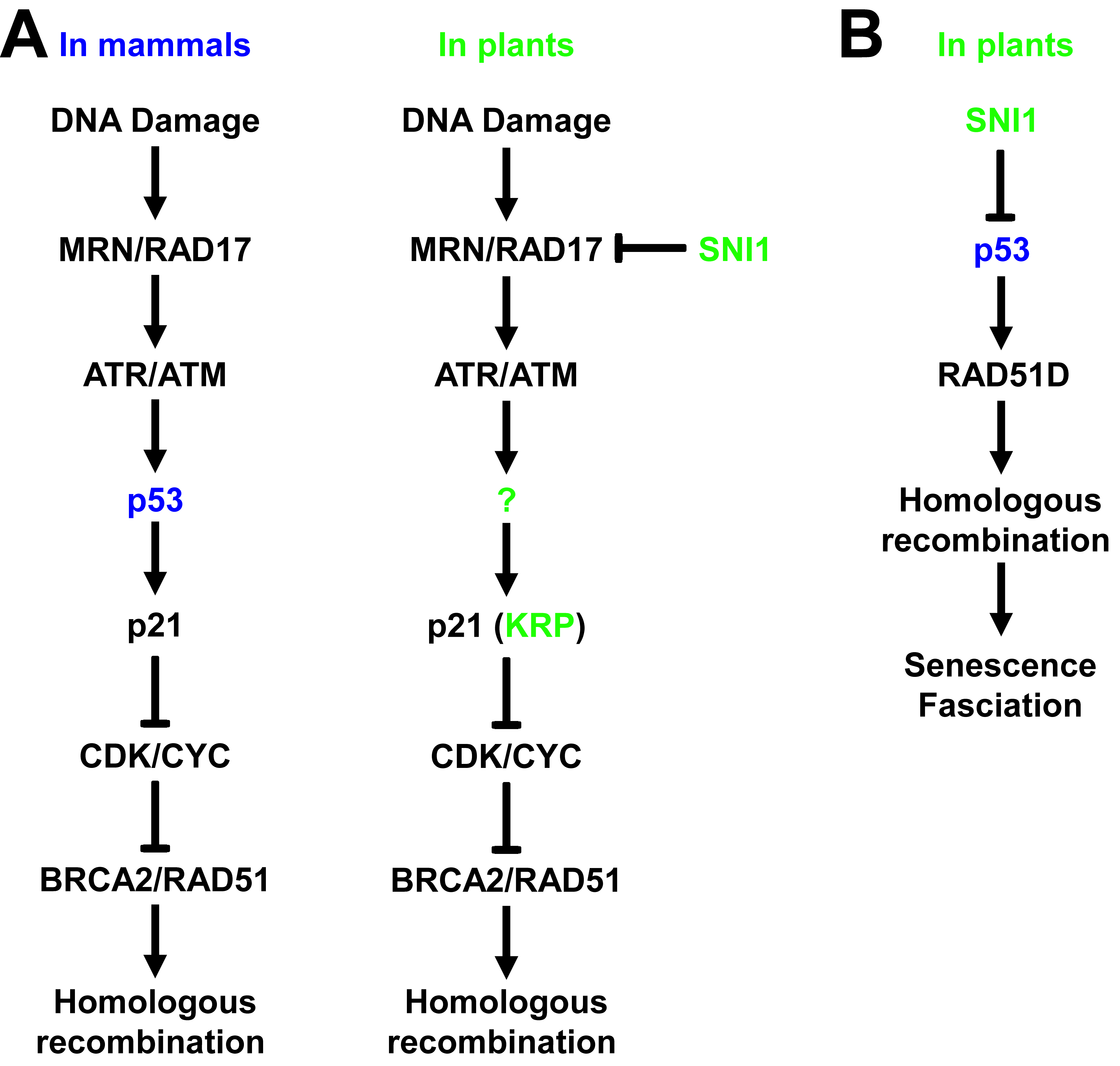

Supplement: S4 Fig — (A) The signaling pathway of DNA damage repair through homologous recombination in mammals and plants. In mammals, the MRE11/RAD50/NBS1 (MRN) complex functions as double strand brake (DSB) sensors. RAD17 recruits the MRN complex to the DSB site. In response to DNA damage, two phosphoinositide 3-kinase-like kinases, ATM and ATR, are activated by the MRN complex to phosphorylate the transcription factor p53. p21, a target of p53, is an inhibitor of cyclin-dependent kinase (CDK). KIP-RELATED PROTEIN (KRP) is the homolog of p21 in plant. CDK phosphorylates BREAST CANCER 2 (BRCA2) which is a mediator of the recombinase RAD51. BRCA2 first loads RAD51 on the DSB site. After the DSB is repaired by RAD51, BRCA2 is then phosphorylated by CDK to remove RAD51 from DNA. Plants possess the homologs of these DNA repair proteins, except for p53. On the contrary, mammals do not bear SNI1. SNI1 is a negative regulator of homologous recombination in plants. Although genetic study reveals that SNI1 functions upstream of ATR, RAD17, BRCA2, and RAD51, how SNI1 is regulated remains unknown. (B) A proposed model of p53 action in plant. p53 induces homologous recombination through the SNI1-RAD51D signaling pathway in plant, leading to senescence and fasciation. (TIF) [file pone.0162840.s004.tif]
