# Supplementary material for: Influence of Human p53 on Plant Development
Source: PLoS One. 2016 Sep 20;11(9):e0162840. doi: 10.1371/journal.pone.0162840 (PMC5029891; doi:10.1371/journal.pone.0162840)
Supplement: S1 Table — (DOCX) [file pone.0162840.s005.docx]

**S1 Table The sectors of recombined GUS reporter (1445) in wild type and three lines of *35S:p53*-transgenic plants**

| **Sample #** | **WT (1445)** | **p53 (1445)** | | |
| --- | --- | --- | --- | --- |
|  |  | **Line1** | **Line2** | **Line3** |
| 1 | 1 | 2 | 7 | 9 |
| 2 | 2 | 7 | 9 | 4 |
| 3 | 0 | 11 | 12 | 5 |
| 4 | 0 | 6 | 3 | 12 |
| 5 | 3 | 8 | 4 | 5 |
| 6 | 1 | 2 | 0 | 3 |
| 7 | 0 | 13 | 5 | 2 |
| 8 | 0 | 5 | 7 | 7 |
| 9 | 0 | 1 | 4 | 3 |
| 10 | 2 | 14 | 9 | 6 |
| 11 | 0 | 3 | 9 | 7 |
| 12 | 2 | 4 | 6 | 8 |
| 13 | 3 | 0 | 11 | 0 |
| 14 | 2 | 14 | 4 | 12 |
| 15 | 4 | 8 | 3 | 3 |
| 16 | 3 | 6 | 7 | 5 |
| 17 | 6 | 9 | 2 | 6 |
| 18 | 2 | 5 | 12 | 7 |
| 19 | 0 | 12 | 4 | 13 |
| 20 | 0 | 2 | 3 | 4 |
| 21 | 3 | 9 | 6 | 8 |
| 22 | 4 | 10 | 3 | 2 |
| 23 | 0 | 4 | 9 | 9 |
| 24 | 0 | 6 | 4 | 3 |
| 25 | 4 | 7 | 8 | 12 |
| 26 | 6 | 4 | 2 | 8 |
| 27 | 1 | 7 | 5 | 9 |
| 28 | 0 | 8 | 2 | 2 |
| 29 | 2 | 9 | 9 | 7 |
| 30 | 0 | 6 | 11 | 8 |
| 31 | 1 | 2 | 4 | 3 |
| 32 | 0 | 11 | 3 | 12 |
| 33 | 2 | 2 | 4 | 7 |
| 34 | 1 | 4 | 0 | 11 |
| 35 | 0 | 5 | 7 | 6 |
| 36 | 0 | 4 | 5 | 6 |
| 37 | 1 | 5 | 0 | 1 |
| 38 | 1 | 2 | 12 | 7 |
| 39 | 0 | 8 | 5 | 8 |
| 40 | 3 | 0 | 5 | 8 |
| 41 | 2 | 16 | 7 | 6 |
| 42 | 3 | 3 | 3 | 9 |
| 43 | 1 | 2 | 9 | 4 |
| 44 | 0 | 4 | 8 | 3 |
| 45 | 0 | 2 | 4 | 0 |
| 46 | 1 | 7 | 3 | 12 |
| 47 | 2 | 6 | 9 | 9 |
| 48 | 0 | 1 | 2 | 5 |
| 49 | 4 | 9 | 8 | 7 |
| 50 | 0 | 4 | 5 | 5 |
| 51 | 0 | 2 | 3 | 4 |
| 52 | 3 | 8 | 2 | 8 |
| 53 | 2 | 4 | 7 |  |
| 54 | 0 | 9 | 8 |  |
| 55 | 4 | 2 | 2 |  |
| 56 | 3 | 2 | 7 |  |
| 57 | 0 | 0 | 7 |  |
| 58 | 2 | 13 | 1 |  |
| 59 | 3 | 11 | 9 |  |
| 60 | 0 | 2 | 2 |  |
| 61 | 0 | 5 | 4 |  |
| 62 | 4 | 6 | 12 |  |
| 63 | 3 | 7 | 7 |  |
| 64 | 2 | 15 | 1 |  |
| 65 | 0 | 12 | 8 |  |
| 66 | 2 | 0 | 0 |  |
| 67 | 2 | 3 | 9 |  |
| 68 | 1 | 4 | 12 |  |
| 69 | 0 | 13 | 4 |  |
| 70 | 3 | 7 | 2 |  |
| 71 | 2 | 9 | 13 |  |
| 72 | 3 | 6 | 3 |  |
| 73 | 3 | 6 |  |  |
| 74 | 2 | 5 |  |  |
| 75 | 3 | 3 |  |  |
| 76 | 2 | 0 |  |  |
| 77 | 1 | 7 |  |  |
| 78 | 3 | 3 |  |  |
| 79 | 0 | 11 |  |  |
| 80 | 2 | 8 |  |  |
| 81 | 2 | 9 |  |  |
| 82 | 0 | 7 |  |  |
| 83 | 3 | 7 |  |  |
| 84 | 0 | 12 |  |  |
| 85 | 0 | 3 |  |  |
| 86 | 4 | 0 |  |  |
| 87 | 0 | 8 |  |  |
| 88 | 0 | 2 |  |  |
| 89 | 0 | 3 |  |  |
| 90 | 4 | 11 |  |  |
| 91 | 3 | 13 |  |  |
| 92 | 0 | 15 |  |  |
| 93 | 3 |  |  |  |
| 94 | 0 |  |  |  |
| 95 | 2 |  |  |  |
| 96 | 4 |  |  |  |
| 97 | 3 |  |  |  |
| 98 | 0 |  |  |  |
| 99 | 0 |  |  |  |
| 100 | 3 |  |  |  |
| 101 | 0 |  |  |  |
| 102 | 3 |  |  |  |
| 103 | 3 |  |  |  |
| n | 103 | 92 | 72 | 52 |
| Average | 1.601942 | 6.217391 | 5.638889 | 6.346154 |
| Standard error | 0.151356 | 0.424643 | 0.402606 | 0.454477 |
