# Supplementary material for: Influence of Human p53 on Plant Development
Source: PLoS One. 2016 Sep 20;11(9):e0162840. doi: 10.1371/journal.pone.0162840 (PMC5029891; doi:10.1371/journal.pone.0162840)
Supplement: S2 Table — (DOCX) [file pone.0162840.s006.docx]

**S2 Table The summary of quality control of microarray**

| **Sample** | WT | | | p53 | | |
| --- | --- | --- | --- | --- | --- | --- |
|  | Repeat 1 | Repeat 2 | Repeat 3 | Repeat 1 | Repeat 2 | Repeat 3 |
| **Threshold Test** | Outside Bounds | Outside Bounds | Outside Bounds | Outside Bounds | Outside Bounds | Outside Bounds |
| **TGT** | 250 | 250 | 250 | 250 | 250 | 250 |
| **RawQ** | 2.486434 | 2.5 | 2.320168 | 2.288531 | 2.357702 | 2.349572 |
| **Background (BG) Avg** | 62.535816 | 60 | 54.079082 | 54.80319 | 56.33629 | 54.59766 |
| **Noise Avg** | 2.920787 | 2.9 | 2.557617 | 2.627289 | 2.723179 | 2.616655 |
| **%P** | 62.240246 | 62 | 61.06094 | 62.27532 | 63.22665 | 60.11399 |
| **Spike_AFFX-r2-Bs-dap_3-5-ratio** | 0.855174 | 0.5 | 2.061253 | 0.273361 | 0.11744 | 1.045412 |
| **Spike_AFFX-r2-Bs-lys_3-5-ratio** | 2.666729 | 4.2 | 8.322852 | 2.198114 | 3.200074 | 3.724997 |
| **Spike_AFFX-r2-Bs-phe_3-5-ratio** | 2.313256 | 0.2 | 1.26218 | 11.8355 | 0.506959 | 26.95363 |
| **Spike_AFFX-r2-Bs-thr_3-5-ratio** | 0.417486 | 0.1 | 2.193528 | 0.181109 | 0.154876 | 1.555194 |
| **Spike_AFFX-r2-Ec-bioB_3-5-ratio** | 0.603182 | 0.8 | 0.777479 | 1.109706 | 0.815928 | 1.190499 |
| **Spike_AFFX-r2-Ec-bioC_3-5-ratio** | 1.116107 | 1.1 | 1.228103 | 1.029622 | 1.082247 | 1.085311 |
| **Spike_AFFX-r2-Ec-bioD_3-5-ratio** | 1.36376 | 1.4 | 1.317744 | 1.260556 | 1.27857 | 1.318729 |
| **Spike_AFFX-r2-P1-cre_3-5-ratio** | 1.269338 | 1.3 | 1.268133 | 1.292052 | 1.242694 | 1.244455 |
