# Supplementary material for: Influence of Human p53 on Plant Development
Source: PLoS One. 2016 Sep 20;11(9):e0162840. doi: 10.1371/journal.pone.0162840 (PMC5029891; doi:10.1371/journal.pone.0162840)
Supplement: S3 Table — (DOCX) [file pone.0162840.s007.docx]

**S3 Table Quality assay for reverse transcription and qPCR**

**Quality assay for reverse transcription:** Five μg of RNA was treated with DNase (Ambion TURBO DNA-free Kit, Thermo Fisher). Two μg of DNase-treated RNA was used to synthesize cDNA using the SuperScript III cDNA Synthesis (Invitrogen). The synthesized cDNAs were diluted 5 times. The transcripts of *UBQ5* in three batches of reverse transcriptions were detected by qPCR. The standard deviation was < 0.25 (95% probability), indicating the reverse transcription is reproducible. The statistical analysis (one-way ANOVA with Bonferroni post hoc test) showed that there was no significant difference between three replicates (p < 0.01).

| ***UBQ5*** | | | | |
| --- | --- | --- | --- | --- |
| Reverse transcription | | | | |
| Repeat 1 | Repeat 2 | Repeat 3 | Mean | Standard deviation |
| 16.67 | 17.08 | 16.75 | 16.83 | 0.22 |

**Serial dilutions of reverse transcription product (cDNA) were used for quality assay of qPCR for *UBQ5* and *RAD51D*.**

| *UBQ5* (C_t_) | | | | | |
| --- | --- | --- | --- | --- | --- |
| Dilution | Repeat 1 | Repeat 2 | Repeat 3 | Mean | Standard deviation |
| 1X | 14.767 | 14.979 | 14.848 | 14.865 | 0.107 |
| 3X | 15.998 | 16.129 | 16.377 | 16.168 | 0.192 |
| 9X | 17.599 | 17.593 | 17.580 | 17.591 | 0.010 |
| 27X | 19.336 | 19.115 | 19.296 | 19.249 | 0.118 |
| 81X | 21.349 | 20.911 | 21.294 | 21.185 | 0.238 |
| 243X | 22.930 | 22.681 | 22.866 | 22.826 | 0.130 |
| y = - 3.3842x + 32.836, R² = 0.9955 | | | | | |
|  | | | | | |
| *RAD51D* (C_t_) | | | | | |
| Dilution | Repeat 1 | Repeat 2 | Repeat 3 | Mean | Standard deviation |
| 1X | 17.331 | 17.223 | 17.398 | 17.317 | 0.089 |
| 3X | 18.861 | 19.104 | 19.270 | 19.079 | 0.206 |
| 9X | 20.762 | 20.550 | 20.823 | 20.712 | 0.144 |
| 27X | 22.150 | 22.503 | 22.210 | 22.288 | 0.188 |
| 81X | 24.562 | 24.169 | 24.249 | 24.327 | 0.208 |
| 243X | 25.789 | 25.964 | 25.537 | 25.763 | 0.215 |
| y = - 3.5661x + 36.533, R² = 0.9987 | | | | | |

**Comparison of the key parameters of qPCR assay**

Amplification = 10 ^(-1/slope)^

Efficiency = [10 ^(-1/slope)^] – 1

| Gene | Slope | Amplification | Efficiency |
| --- | --- | --- | --- |
| *UBQ5* | -3.3842 | 1.9747 | 0.9747 |
| *RAD51D* | -3.5661 | 1.9073 | 0.9073 |

**Conclusion**: The quality assessments showed that the qPCR assays for *UBQ5* and *RAD51D* are comparable as indicated by the key parameters of the qPCR reaction including slope (log-linear phase of the amplification reaction, -3.1 ~ -3.6), R2 (> 0.99), precision (standard deviation < 0.167, 99.7% probability; standard deviation < 0.25, 95% probability), amplification (~ 2.0) and efficiency (0.9 ~ 1.1). The numbers in the brackets are considered to be in the range of good quality.
